# Quasi-cluster randomized trial of a six-month low-intensity group-based resistance exercise for hemodialysis patients on depression and cognitive function: a 12-month follow-up

**DOI:** 10.1080/21642850.2021.1966302

**Published:** 2021-08-30

**Authors:** Nanako Nakamura-Taira, Naoshi Horikawa, Fumie Oka, Yuri Igarashi, Sayaka Kobayashi, Shingo Kato, Takashi Enomoto, Hitomi Kimura, Yukari Watanabe, Toru Kumada, Kimihiko Matsuyama, Naoki Matsuoka, Haruo Yoshimasu

**Affiliations:** a Department of Psychology, Faculty of Letters, Chuo University, Tokyo, Japan; b Department of Psychiatry, Saitama Medical Center, Saitama Medical University, Saitama, Japan; c Department of Psychiatry and Huntsman Mental Health Institute, University of Utah, UT, USA, UT, USA; d Department of Psychological Counseling, Faculty of Humanities, Tokyo Kasei University, Tokyo, Japan; e Yanagihara Rehabilitation Hospital, Tokyo, Japan; f Misato Central General Hospital, Saitama, Japan; g Misato Kenwa Clinic, Saitama, Japan; h Chiba Aiyukai Kinen Hospital, Chiba, Japan

**Keywords:** Hemodialysis patients, low-intensity exercise, group resistance exercise, depressive symptoms, cognitive function, behavioral and psychological problems associated with cognitive decline, subjective insomnia, exercise self-efficacy

## Abstract

**Objective:**

This study aimed to examine the effects of a six-month group-based low-intensity resistance exercise program on depression and the cognitive function of hemodialysis patients.

**Method:**

We conducted a quasi-cluster randomized, open-label controlled study from October 2017 to December 2018. Forty-two patients undergoing hemodialysis completed the trial over six months; half participated in the resistance exercise group (*n* = 21, mean = 74.90 years of age, *SD* = 2.23, 66.67% female) and the other half were in a stretching control group (*n* = 21, mean = 72.57 years of age, SD = 2.26, 28.57% female). Depressive symptoms and cognitive function were the primary outcome measures. Behavioral and psychological problems associated with cognitive decline (NPI-Q), subjective insomnia, and exercise self-efficacy were secondary outcomes. Outcomes were measured at baseline, three-month (mid-intervention), six-month (end of intervention), and 12-month (six months after intervention) follow-ups. Linear mixed model analyses were used to determine short-term (immediately after intervention) and long-term (six months after intervention) effects.

**Results:**

In depression, cognitive function, and the NPI-Q, there were no significant effects. In subjective insomnia, a short-term group-by-time interaction in the intervention group compared to the control group was found (ES = .43). However, the effect had disappeared by the 12-month follow-up. In exercise self-efficacy, short- and long-term group-by-time interactions were found. A significant short-term increase in the resistance exercise and a significant decrease in the stretching control was observed (ES = -.83). However, the effect was weakened in the long term (ES = -.38).

**Conclusion:**

The results showed that low-intensity group resistance exercise would reduce subjective insomnia and improve exercise self-efficacy, but the effect was not maintained by six months after the program.

**Trial registration:** This study was registered on the University Hospital Medical Information Network Clinical Trials Registry (UMIN000029372).

**Trial registration:** UMIN Japan identifier: UMIN000029372.

## 1. Introduction

Hemodialysis patients are known to experience a variety of psychosomatic symptoms; depression and cognitive decline are frequently observed. According to a meta-analysis (Palmer et al., 2013), the prevalence of depression based on a diagnostic interview in patients with stage 5D end-stage renal failure was 22.8% (95% confidence interval (CI), 18.6–27.6) and based on a self-administered questionnaire was 39.3% (95% CI 36.8–42.0). In recent years, attention has been focused on cognitive decline in dialysis patients, and the prevalence of cognitive dysfunction has been reported to be 16–38% (Kurella & Yaffe, 2011). Although the mechanisms of depression and cognitive decline in dialysis patients are not clear, evidence suggests that psychosocial and biological factors are interconnected (Farrokhi, Abedi, Beyene, Kurdyak, & Jassal, 2014; Kurella & Yaffe, 2011; Palmer et al., 2013; Patel, Dasgupta, Tadros, & Baharani, 2016; Shirazian et al., 2016).

In psychiatric patients not on dialysis, exercise therapy has been shown to improve depressive symptoms (Cooney et al., 2013; Craft & Perna, 2004; Kunugi, Urushibara, & Nanko, 2004; Paluska & Schwenk, 2000), cognitive function (Heyn, Johnson, & Kramer, 2008; Ströhle et al., 2015), sleep (Kovacevic, Mavros, Heisz, & Singh, 2018), and behavioral and psychological problems associated with cognitive decline (Barreto, Demougeot, Pillard, Lapeyre-Mestre, & Rolland, 2015; Kredlow, Capozzoli, Hearon, Calkins, & Otto, 2015; Potter, Ellard, Rees, & Thorogood, 2011; Thuné-Boyle, Iliffe, Cerga-Pashoja, Lowery, & Warner, 2012). In the context of dialysis, recent studies on exercise therapy demonstrated the effectiveness not only for physical impairment (Matsuzawa et al., 2017) but also for alleviating psychological problems, such as depressive symptoms (Chung, Yeh, & Liu, 2017; Shimoda et al., 2017) and insomnia (Amini, Goudarzi, Masoudi, Ahmadi, & Momeni, 2016; Maniam et al., 2014).

Exercise therapy during dialysis with regard to depressive symptoms and cognitive function leaves two issues to be addressed. The first is the effects of exercise modalities, except for middle-high intensity resistance exercise that have been examined in previous studies. Given the growing demand for exercise training programs for hemodialysis patients that are feasible, safe, and require minimal staff time, we investigated the effects of group-based own-weight exercise training (Aida et al., 2007; Benavent-Caballer, Rosado-Calatayud, Segura-Ortí, Amer-Cuenca, & Lisón, 2016; Kanamori, Takamiya, & Inoue, 2015) on depression and cognitive function.

We focused on low-intensity resistance exercise. In previous studies, moderate or higher-intensity aerobic exercise during dialysis (e.g. riding a bicycle ergometer on a dialysis bed) significantly improved depressive symptoms (Chung et al., 2017; Shimoda et al., 2017). However, the effects of other exercise modalities (e.g. resistance training) and intensities (e.g. low intensity) have not been fully investigated (Chung et al., 2017; Shimoda et al., 2017). Therefore, we investigated the effect of the group-based low-intensity resistance exercise on depression of dialysis patients. This type of exercise has another benefit. High-intensity and individual exercise programs have limited feasibility (Chen et al., 2010; Mura & Carta, 2013), especially given that many Japanese dialysis patients are older and experience difficulties performing these types of exercise. An increasing number of older dialysis patients are prone to geriatric problems, such as frailty (Johansen, Chertow, Jin, & Kutner, 2007; Kurella, Covinsky, Collins, & Chertow, 2007). In addition, dialysis centers do not have sufficient staff to run individualized exercise programs (Sakurai et al., 2017). Therefore, group-based low-intensity exercise would be beneficial and feasible for patients and medical staff.

We also investigated the effect of resistance exercises on the cognitive function of dialysis patients. Questionnaire studies of dialysis patients have investigated the relationship between physical activity level in daily life and cognitive function (Bronas, Puzantian, & Hannan, 2017; Stringuetta-Belik et al., 2012). Several intervention studies on cognitive function (Chu & McAdams-DeMarco, 2019) have also been carried out, but the current number of studies is inadequate (Chu & McAdams-DeMarco, 2019; Kaltsatou et al., 2015; Patel et al., 2016; Stringuetta-Belik et al., 2012). Although the mechanisms of the effectiveness of the exercise on cognitive functions are not clear, evidence suggests that physical, psychological, and lifestyle pathways contribute (Chu & McAdams-DeMarco, 2019).

Furthermore, we measured a six-month follow-up after the program (12 months from baseline). Meta-analysis (Bessa et al., 2015; Song, Hu, Diao, Chen, & Jiang, 2018) showed the effectiveness of the exercise program for dialysis patients, but most of the studies measured only the aftermath of the program. Therefore, research that investigates long-term effect is needed.

We hypothesized that a group-based resistance exercise program would alleviate depression and preserve cognitive function among hemodialysis patients in general hospital settings. We investigated the efficacy of this approach in two dialysis centers, with depressive symptoms and cognitive function as the primary outcomes. We also measured behavioral and psychological problems associated with cognitive decline, sleep problems, and exercise self-efficacy as secondary outcomes. Although depression and sleep problems are prevalent in dialysis patients (Paparrigopoulos & Theleritis, 2009; Rodriguez et al., 2013), the relationship between them is unclear (Maung et al., 2017). Exercise self-efficacy was also measured because we assumed that this variable would be enhanced through participation in the exercise program (Bandura, 1982; Maeda, Shen, Schwarz, Farrell, & Mallon, 2013).

A quasi-cluster randomization was chosen for practical reasons and due to the nature of the group-based program. The main purpose of the present study was to use a quasi-cluster randomized trial in an evaluation comparing the effectiveness of different approaches to alleviate depression or promote cognitive function in group-based resistance exercise. The outcomes were at the level of individual participants and measured baseline, three-month (mid-intervention), six-months (post-intervention), and 12-month (six months after intervention) follow-ups.

## 2. Methods

### 2.1. Patient population and recruitment

Participants were recruited from the Misato Kenwa Clinic and Misato Central General Hospital. The eligibility criteria were the following: aged 20 years or older, hemodialysis outpatient. The participants were provided with written informed consent individually. The exclusion criteria were the following: contraindications for exercise training per the Japanese Guidelines for Rehabilitation in Patients with Cardiovascular Disease (Nohara et al., 2014) and Japanese Guideline for Renal Rehabilitation (Yamagata et al., 2019), or diagnosis of severe mental disorders or major neurocognitive disorder.

### 2.2. Trial design

The study was a two-center, quasi-cluster randomized, open-label, controlled clinical trial conducted in Japan at the Misato Kenwa Clinic and Misato Central General Hospital from October 2017 to December 2018. The study protocol was approved by the ethics committee at the Misato Kenwa Clinic and Misato Central General Hospital and written informed consent was obtained from all participants. This study was registered with the University Hospital Medical Information Network Clinical Trials Registry (www.umin.ac.jp/ctr/) as UMIN000029372.

### 2.3. Randomization

The quasi-randomization was carried out after the baseline measurement was conducted. A cluster was formed by an exercise group of patients undergoing dialysis in the same dialysis room on the same morning shift (Dziubek et al., 2016). There were four clusters (two hospitals with two groups each), and participants in each group had the same dialysis schedule (thrice per week: Monday-Wednesday-Friday or Tuesday-Thursday-Saturday). In each hospital, medical staff (who were not research staff) performed an alternative allocation method according to the order of the dialysis schedule. The clusters were assigned the resistance exercise (intervention: Monday-Wednesday-Friday) or stretching (control: Tuesday-Thursday-Saturday) group. Given the nature of the intervention, the study was open-label and unblinded. The research staff was not directly involved in the allocation process.

### 2.4. Study conditions

All participants received their usual medical treatment. If participants were already taking a psychotropic drug (benzodiazepine, donepezil), they continued to receive the same agent during the study. The six-month intervention comprised intradialytic stretching and resistance exercise sessions. All program components were conducted in a group format and from a supine position on the dialysis bed.

The exercise content was prepared by exercise and rehabilitation specialists, and the Borg rating of perceived exertion (RPE; Borg, 1970; Borg, 1982) was expected to be 11–13 (easy to moderate). Both the intervention exercise and control stretching was performed on the bed without any equipment. The instructor provided verbal instruction on how to perform the exercises during the session. The participants followed the verbal instructions and did not receive additional interventions, such as written instructions (e.g. an exercise leaflet).

The intervention and control group participated in the program thrice a week during morning dialysis for six months under the guidance of a rehabilitation expert. For safety, one or two medical staff in addition to the instructor who observed the patients. They also supported the instructor (e.g. correcting patients if they made wrong movements). Since exercise performed during the first half of the period when circulatory dynamics are relatively stable is believed to be useful, the exercise was performed about one hour after the start of dialysis (Chung et al., 2017; Kaltsatou et al., 2015; Shimoda et al., 2017; Stringuetta-Belik et al., 2012).

In both groups, participation was discontinued in accordance with renal rehabilitation criteria (Dziubek et al., 2016; Yamagata et al., 2019). Participants who expressed unwillingness to continue were free to withdraw. If a physician determined that participation should be interrupted or stopped for any patient, they were considered dropouts for the participation in each session.

#### 2.4.1. Resistance exercise intervention

In the intervention group, after about five minutes of warm-up stretching, the resistance exercise began and consisted of lower-limb resistance exercise for 15–20 min. Exercise repetition time and therefore total exercise time was increased every two months so that the intensity of the exercise was maintained despite any training effects. After the exercise, participants performed cool-down stretching for about five minutes. The total program time was 25–30 min. The program settings had some behavior change components (Michie et al., 2013), including instruction on how to perform a behavior, monitoring outcomes of behavior by others without feedback, practical social support, unspecified social support, social comparison, prompts/cues, behavioral practice, habit formation, and graded tasks.

#### 2.4.2. Stretching control

The control group performed only stretching to control any effects that might be attributable to performing something under instruction (Dunn, Trivedi, Kampert, Clark, & Chambliss, 2005) during dialysis. A meta-analysis examining the effects of exercise programs on depression showed that control groups were not expected to improve physical function as a placebo; therefore, rather than no intervention, it was recommended that control groups engage in unloaded exercise (Stubbs et al., 2016). The control group performed warm-up and cool-down stretching for about five minutes each, similar to the intervention group, but without the resistance exercise between stretching sessions.

### 2.5. Measures

#### 2.5.1. Exercise implementation rate

The Borg RPE measures the exercise intensity felt by a person on a scale of 6–20, where 7 = *very, very light* (30% effort), 9 = *very light* (50% effort), 11 = *fairly light* (60% effort), 13 = *somewhat hard* (70% effort), 15 = *hard* (80% effort), 17 = *very hard* (90% effort), and 19 = *very, very hard* (100% effort). Medical staff confirmed the RPE (Borg, 1970) with the participants during exercise.

The program was held on the participants’ beds in the usual dialysis room, and all the participants could naturally attend the program except for those absent. However, the participants’ attitudes toward the exercise had been presumed to vary. Therefore, staff members who observed the program, rated each patient’s participation status in each session (0: *no participation*, 1: *partial participation according partially to the instruction*, 2: *sufficient participation according fully to the instruction*).

At the end of the exercise program (at six months), participants provided their impression of the exercise program as 1: *negative*, 2: *neutral* (e.g. neither good nor bad, nothing special), or 3: *positive*.

#### 2.5.2. Primary outcomes

The Japanese version of the Patient Health Questionnaire-9 (PHQ-9) (Kroenke, Spitzer, & Williams, 2001; Muramatsu et al., 2007; Muramatsu et al., 2018) is a self-rated measure of depression symptoms in patients with physical illness, based on the Diagnostic and Statistical Manual of Mental Disorders, Fourth Edition criteria for major depressive disorder. The PHQ-9 has good psychometric properties and is sensitive to treatment-related change. The nine-item scale yields a total score ranging from 0 to 27, where higher scores indicate greater depression severity. The depression cutoff score is 9/10 (Kroenke et al., 2001).

The Japanese version of the Montreal Cognitive Assessment (MoCA-J) (Fujiwara et al., 2010; Nasreddine et al., 2005) is a brief (10–15-minute) screening tool assessing cognitive functions　suitable for assessing mild cognitive impairment. The total score ranges from 0 to 30 points, with higher scores indicating higher cognitive function. The MoCA’s validity as a screen for cognitive impairment in hemodialysis patients was compared to the Mini-Mental State Examination (Ciesielska et al., 2016) and it was used as a measure of the longitudinal change in the patients’ cognitive function (Iyasere, Okai, & Brown, 2017). The MoCA-J’s cutoff score for cognitive impairment is 25/26.

#### 2.5.3. Secondary outcomes

The Japanese version of the Athens Insomnia Scale (AIS) (Okajima, Nakajima, Kobayashi, & Inoue, 2013; Soldatos, Dikeos, & Paparrigopoulos, 2000) was used to assess subjective insomnia. The AIS comprises eight items with scores ranging from 0 to 24. Higher scores on two factors, nighttime sleep problems, and daytime social dysfunction, indicate greater sleep problem severity. The cutoff score for sleep problems is 6/7.

The Japanese version of the Neuropsychiatric Inventory-Brief Questionnaire Form (NPI-Q) (Cummings et al., 1994; Kaufer et al., 2000; Matsumoto et al., 2006) was used to evaluate psychiatric and behavioral problems, with higher scores indicating greater severity. Medical staff evaluated each patient for neuropsychiatric symptom severity and distress. The total NPI-Q severity score represents the sum of the staff ratings of patients’ individual symptom scores (0–30) and the total NPI-Q distress score represents the sum of the staff’s distress scores (0–50).

Exercise self-efficacy (Oka, 2003) was measured. Considering the daily life of the dialysis patient, the original definition of exercise — ‘at least 20–30 min at a time two to three times a week’ — was modified to ‘exercise at least 10 min at a time other than dialysis time at least once a week.’ The original self-reported measure contained five items scored on a five-point scale (0–4); we excluded two items (e.g. ‘I am confident about exercising even when I am busy and have no time’) because almost all the participants were unemployed. Therefore, we used the total value of the three remaining items (score range 0–12), where higher scores indicated more confidence in exercising in daily life despite difficult situations (e.g. bad weather).

### 2.6. Data collection

All outcome measures represented the change in depression and cognitive function scores from baseline to three, six, and 12 months. The assessments were performed by psychiatrists and clinical psychologists. Since the measurement was performed after confirming the patient’s intention to participate in each time point of the measurement, the amount of data recorded at each time point was different.

### 2.7. Statistical analysis

#### 2.7.1. Power analysis

To our knowledge, as no previous study has examined the effects of an exercise program using the same scales as those used to measure primary outcomes in this study, it was difficult to calculate the sample size based on existing research (estimated standard deviation (*SD*) of post-intervention). Therefore, based on Cohen’s calculation method, the sample size was determined from the viewpoint of power analysis. Using G*Power 3.1.9.7 (Faul, Erdfelder, Buchner, & Lang, 2009; Faul, Erdfelder, Lang, & Buchner, 2007), we set an anticipated effect size of 0.30 (Herring, Puetz, O’Connor, & Dishman, 2012), type I error probability = 5%, 80% power, two groups, and four-time repetitions, correlation among repeated measures = 0.5, nonsphericity correction = 0.5, using a repeated-measures analysis of variance within-between subject interactions; the resulting sample size was 28 participants. In cluster randomization, it is necessary to multiply the design effect (Campbell, Piaggio, Elbourne, & Altman, 2012; Ukoumunne et al., 1999). The design effect of 1.28 (calculated with the number of participants in the cluster = 15 and intraclass correlation coefficients = 0.02) was multiplied by 28 to obtain a value of 35.84, and the dropout rate was estimated to be 20%, resulting in a total sample size of 44.8. Therefore, recruitment was conducted with the goal of having 22 participants in each group.

#### 2.7.2. Data analysis

All eligible participants who were quasi-randomly allocated to the two conditions were included in the analysis independent of compliance with the protocol, using an intention-to-treat approach. Significance was accepted at *p *< .05. All calculations were conducted using Stata version 14 (Stata Corp., College Station, Texas, USA).

Baseline comparisons of demographic and other variables between the intervention and control condition were performed using independent two-sample *t*-tests for continuous variables and chi-square tests for categorical variables.

Group (resistance exercise and stretching control) and time (baseline and three-, six-, and 12-month follow-ups) were analyzed using a linear mixed effects model with restricted maximum likelihood (REML). The full model included treatment and time as well as their respective two-way interactions as fixed effect and participant as a random effect. The fixed effect parameters included group, gender, body mass index at baseline because our program used own weight, and time. The random effect for each patient was included in the models to account for the additional component of variance. We conducted two separate mixed model analyses for each outcome measure to describe between short- and long-term intervention effects (Arrogi, Schotte, Bogaerts, Boen, & Seghers, 2017). Three-time points (baseline, three months, and six months) were used as short-term, and four-time points (baseline, three months, six months, 12 months) were used as long-term intervention effects.

For both the intervention and control group, the effect size (Hedge’s *g*) was calculated for changes from baseline to three months (middle in the program), six months (just after the program), and 12 months follow-ups (six months after the program.

## 3. Results

### 3.1. Baseline characteristics

**Figure 1.**
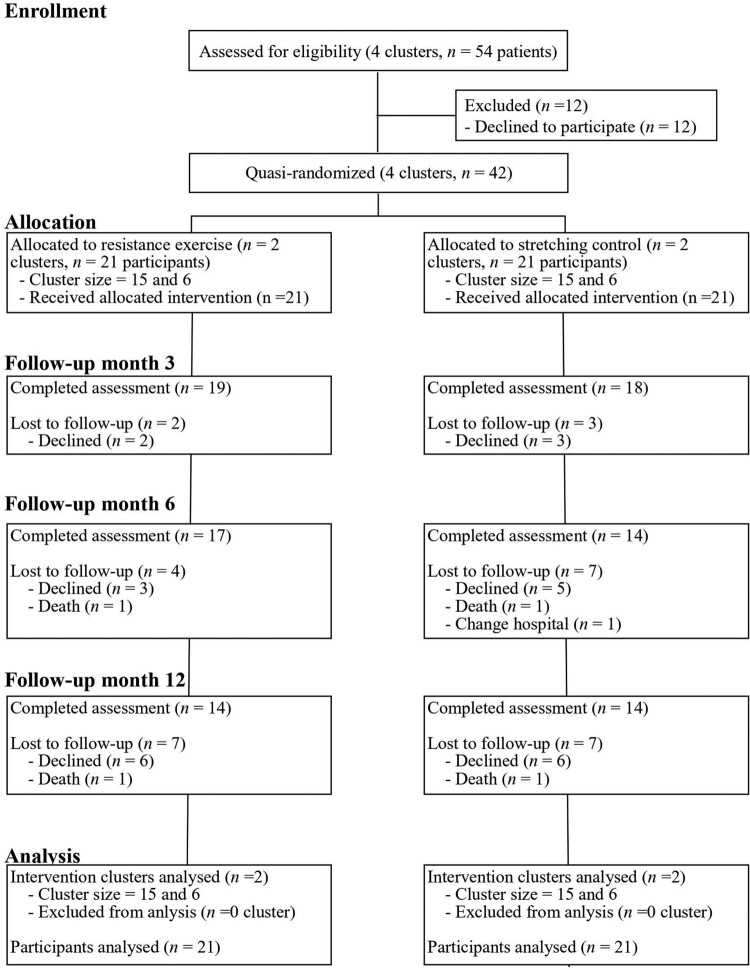
Participant flow diagram.

The participant flowchart is shown in Figure 1. Of 54 patients from four clusters meeting the eligibility criteria, 42 agreed to participate in the study. The four clusters were quasi-randomly divided into two groups, intervention and control; each group had two clusters.

Table 1 shows the participant characteristics at baseline. Except for gender (*χ^2^* (1) = 6.11, *p* = .01), there were no significant differences between the intervention and control groups.

**Table 1.** Baseline characteristics of the participants (*N* = 42).

Baseline characteristic	Resistance exercise group (*n* = 21)		Stretching control group (*n* = 21)		*p*
Age, *years*					.47^a^
Mean (*SD*)	74.90	(2.23)	72.57	(2.26)	
Gender, *n* (%)					.**01**^b^
Male	7	(33.33)	15	(71.43)	
Female	14	(66.67)	6	(28.57)	
Length of dialysis, months					.31^a^
Mean (*SD*)	90.52	(19.16)	119.62	(21.00)	
Education, years					.72^a^
Mean (*SD*)	10.71	(0.55)	10.43	(0.55)	
Employment status, *n* (%)					.55^b^
Unemployed or retired	21	(100.00)	20	(95.24)	
Others	0	(0.00)	1	(4.76)	
Marital status, *n* (%)					.77^b^
Married	10	(47.62)	9	(42.86)	
Single (widowed, etc.)	11	(52.38)	12	(57.14)	
Living status, *n* (%)					.43^b^
Living alone	5	(23.81)	3	(14.29)	
Cohabiting	16	(76.19)	18	(85.71)	
Cause of ESRD, *n* (%)					.35^b^
Diabetic nephropathy	12	(57.14)	11	(52.38)	
Others	8	(38.10)	6	(28.57)	
Cause unknown	1	(4.76)	4	(19.05)	
Body mass index					.66^a^
Mean (*SD*)	21.41	(3.71)	20.92	(3.29)	
Comorbidities^c^, *n* (%)					.92^b^
0	6	(28.57)	5	(23.81)	
1–2	9	(42.86)	9	(42.86)	
> 3	6	(28.57)	7	(33.33)	
Hematocrit, %					.12^a^
Mean (*SD*)	32.67	(0.55)	34.22	(0.79)	
Hemoglobin, g/dL					.08^a^
Mean (*SD*)	10.55	(0.25)	11.09	(0.25)	

^a^ *p* value is based on *t*-test, ^b^*p* value is based on chi-square test, ^c^comorbidity is expressed as a score for which each of the following conditions contributed at baseline: hypertension, coronary heart disease, chronic heart failure, cerebrovascular disease, hyperlipidemia, cancer. *SD*: standard deviation; ESRD: end-stage renal disease.

### 3.2. Exercise participation status

The mean values for the intervention and control groups were compared with *t*-tests. For each RPE, no significant difference was observed (*t* (39) = −0.23, *p* = .81). The mean RPE of the intervention group was 9.87 (*SD* = 2.25) and that of the control group was 9.73 (*SD* = 2.54).

The degree of exercise participation was calculated by the staff members’ ratings. If a session was rated a one or higher was counted as the completed session. The ratio of the number of completed sessions to the total number of exercise sessions performed was defined as the participation rate. If the participation rate in the group exercise program was 33% or more (Helgadóttir, Hallgren, Ekblom, & Forsell, 2016), we considered the protocol completed, with 17 patients (80.95%) in the intervention group and 18 (85.71%) in the control group. There was no significant difference between the groups with regard to program completion rate (χ2 (1) = 0.17, *p* = .68). In addition, there was no significant between-group difference in the mean RPE of those who completed the program and those who withdrew (*t* (39) = 1.93, *p* = .06). The average RPE for dropouts was 11.47 (*SD* = 0.77), while that for completers was 9.50 (*SD* = 2.46).

Participants’ impressions about the exercise program showed that the intervention group and the control group provided mostly positive responses (64.71%, 35.29%, and 0% were positive, neutral, and negative, respectively, in the intervention group; 53.85%, 38.46, and 7.69% were positive, neutral, and negative, respectively, in the control group). No significant difference was found between groups in the proportion of each response (χ^2^ (1) = 1.47, *p* = .48).

### 3.3. Outcomes

#### 3.3.1. Primary outcomes

Table 2 shows the adjusted mean scores, the short- and long-term intervention effects on the outcome scores, and the effect sizes at baseline and at the three-, six-, and 12-month follow-ups. We found neither short- nor long-term intervention effects for the PHQ-9 or MoCA-J.

**Table 2.** Short- and Long-term intervention effects on outcome measures. Adjusted mean scores (SE) for outcome measures over the study period.

Resistance exercise intervention group				Stretching control group				Short-term intervention effect				Long-term intervention effect		
Baseline	3 months	6 months	12months	Baseline	3 months	6 months	12months	Time	2×3	3 months ES	6 months ES	Time	2×4	12 months ES
AMS (*SE*)	AMS (*SE*)	AMS (*SE*)	AMS (*SE*)	AMS (*SE*)	AMS (*SE*)	AMS (*SE*)	AMS (*SE*)	Z	Z	g [95%CI]	g [95%CI]	Z	Z	g [95%CI]
PHQ-9, score range: 0–27														
5.93 (0.72)	3.61 (0.73)	3.56 (0.80)	3.74 (1.00)	6.67 (0.83)	4.59 (0.87)	5.69 (1.03)	4.58 (0.89)	−1.62	0.79	0.04	0.20	−1.26	0.45	0.01
										[−0.57:0.64]	[−0.41:0.81]			[−0.59:0.62]
MOCA−J, score range: 0−31														
18.45 (0.63)	18.66 (0.64)	18.87 (0.71)	20.69 (0.99)	18.48 (0.77)	18.30 (0.80)	18.09 (0.94)	18.65 (0.91)	0.62	−0.61	−0.07	−0.13	1.57	−1.23	−0.30
										[−0.67:0.54]	[−0.74:0.48]			[−0.91:0.31]
NPI−Q severity, score range: 0−3														
0.56 (0.17)	0.68 (0.17)	0.46 (0.17)	0.51 (0.19)	0.54 (0.17)	0.23 (0.17)	0.28 (0.19)	0.50 (0.18)	0.25	−0.70	−0.33	−0.13	−0.43	0.29	0.01
										[−0.94:0.28]	[−0.74:0.48]			[−0.59:0.62]
NPI−Q distress, score range: 0−5														
0.68 (0.30)	1.05 (0.30)	0.60 (0.31)	0.58 (0.34)	0.69 (0.29)	0.29 (0.31)	0.40 (0.34)	0.53 (0.31)	0.20	−0.42	−0.30	−0.07	−0.34	0.13	−0.03
										[−0.91:0.31]	[−0.68:0.53]			[−0.63:0.58]
Athens Insomnia Scale, score range: 0−24														
5.21 (0.68)	2.34 (0.70)	2.49 (0.76)	4.47 (0.95)	5.77 (0.72)	4.31 (0.76)	6.31 (0.91)	4.42 (0.79)	−1.92	**1**.98*	0.20	0.43	0.88	0.37	−0.08
										[−0.40:0.81]	[−0.18:1.05]			[−0.69:0.52]
Exercise self−efficacy, score range: 0−12														
5.83 (0.82)	5.33 (0.83)	7.93 (0.89)	6.34 (1.17)	5.89 (0.81)	3.37 (0.85)	2.27 (0.99)	3.41 (0.90)	**3**.40**	**−3**.88***	−0.30	−0.83	**2**.37*	**−2**.77**	−0.38
										[−0.91:0.31]	[−1.46:−0.20]			[−0.99:0.23]

AMS: adjusted mean score based on long-term intervention effect, *SE*: standard error, Time: Main effect of time, 2×3: 2 (group)×3 (time) interaction effect estimated with linear mixed model, 3months ES: effect size of difference between groups at 3 months, g: Hedges’ g, 6 months ES: effect size of difference between groups at 6 months, 2×4: 2 (group)×4 (time) interaction effect estimated with linear mixed model, 12 months ES: effect size of difference between groups at 12 months, * *p* < .05, ** *p* < .01, *** *p* < .001.

Note: Bold font indicates significant effect. Scores are adjusted for baseline age and body mass index.

#### 3.3.2. Secondary outcomes

**Figure 2.**
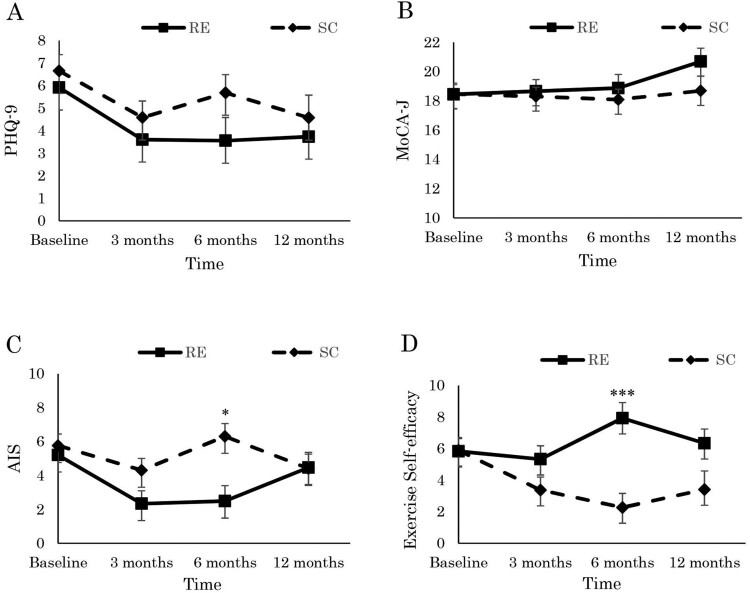
Effects of the low-intensity group resistance exercise on (A) depression, (B) cognitive function, (C) subjective insomnia, (D) exercise self-efficacy in dialysis patients at baseline, 3-, 6-, 12- month. Statistical difference test of significance compared with control group from obtained from linear mixed model. * *p* < .05 interaction, *** *p* < .001 interaction, RE: resistance exercise, SC: stretching control, PHQ-9: Patient Health Questionnaire, MoCA-J: Japanese version of Montreal Cognitive Assessment, AIS: Japanese version of Athens Insomnia Scale. Note: Scores are the adjusted for baseline age and baseline body mass index.

As shown in Table 2 and Figure 2, we found neither short- nor long-term intervention effects for NPI-Q severity or distress. Although the AIS showed a short-term interaction effect (Hedges’s g = 0.43 at six months), it did not show a long-term intervention effect. Exercise self-efficacy showed a short-term group-by-time interaction effect (Hedges’s g = −0.83 at six months) and a long-term group-by-time interaction effect with a small effect size (Hedges’s g = −0.38 at 12 months). The results indicate that the resistance exercise group’s subjective insomnia and self-efficacy scores improved over the intervention period but were not maintained at the 12-month follow-up (Figure 2).

## 4. Discussion

The purpose of this study was to examine the short- and long-term effect of a intradialysis low-intensity group exercise program on depressive symptoms, cognitive function, and related psychological indicators among dialysis patients. The results of a six-month intradialysis low-intensity group resistance exercise program showed the short-term effect on subjective insomnia with medium effect size and showed the short-term effect with large effect size and the long-term effect with small effect size on self-efficacy.

The results showed that low-intensity group resistance exercise was not effective in improving depressive symptoms in dialysis patients. One explanation is the low severity level of the depression of the participants at baseline. In previous studies, the effects of higher-intensity aerobic exercise therapy on depressive symptoms of dialysis patients have been observed for moderate or severe depression (Chung et al., 2017; Shimoda et al., 2017). Previous meta-analysis of the effectiveness of resistance training on depression for no-dialysis patients showed that the mean effect for RCTs in which baseline scores were indicative of mild to moderate depression was significantly larger than from RCTs in which scores were below clinical cutoff scores (Gordon et al., 2018). In this study, the baseline depression score was under the cutoff point. This might have had an insufficient effect on the program.

Neither group showed improvements in cognitive function and behavioral problems associated with cognitive decline. Low-intensity resistance exercise did not improve cognitive function or psychological and behavioral problems associated with cognitive decline in this study’s participants. A meta-analysis (Barreto et al., 2015) of improved psychological and behavioral problems from exercise in patients with dementia pointed out that few studies included in the review reported exercise intensity. Therefore, future studies should include measures of exercise intensity. Although mechanisms that improve cognitive function of dialysis patients (e.g. increasing cerebral blood flow, brain-derived neurotropic factor, reducing inflammatory markers) were suggested by previous research (Chu & McAdams-DeMarco, 2019), our exercise protocol might be of insufficient workload to stimulate positive effects. Additionally, participants’ average cognitive function scores were below the cutoff value in this study, even though patients with a dementia diagnosis were excluded. Cognitive function was assessed during dialysis for practical reasons, which could have affected the results (Shirazian et al., 2016).

The findings showed that low-intensity resistance exercise for a sufficient period, such as six months, can be effective in improving subjective insomnia. While the frequency of sleep disorders in dialysis patients is as high as 65–85% (Pierratos & Hanly, 2011), the effects of exercise therapy in improving subjective insomnia are not consistent (Song et al., 2018; Yang et al., 2015). For example, a meta-analysis of intervention studies in dialysis patients with restless legs syndrome showed that while depressive symptoms were relieved, sleep quality was not significantly improved (Yang et al., 2015). Many of the studies involved aerobic exercises, with subjective exercise intensities (Borg, 1982) ranging from 11 to 16 (easy to very hard). A review of non-pharmacological therapies for subjective insomnia in dialysis patients also indicated that differences in the content of exercise therapy were insufficiently examined (Yang et al., 2015). Therefore, in this study, low-intensity resistance exercises were performed, and significant improvement was observed, providing new knowledge about the intensity of exercise therapy needed to improve subjective insomnia in dialysis patients.

While low-intensity exercise had an effect on subjective insomnia, it was not maintained in the long term. This suggests that regular intradialysis light exercise should be continued to maintain sleep quality. The exercise program implemented in this study did not involve much of a burden for the dialysis patients and medical staff and is, therefore, easy to execute. The effects of continuing such exercise programs for long periods will need to be considered further.

Interesting results were observed for exercise self-efficacy, where short- and long-term group-by-time interactions were observed, and exercise self-efficacy pertaining to exercise in daily life was significantly improved in the intervention group but decreased in the control group at immediately after exercise and follow-up. Exercise self-efficacy as measured in this study reflected confidence about the practice of exercise in daily life. Although this study does not present data regarding improvement in physical function, the exercises may have improved physical function in the intervention group, and thereby increased confidence in performing exercise in daily life. Another possible explanation would be assumed. Some interpersonal interaction between the medical staff and the participants during the group-based program (do Carmo, da Rocha, & Tanaka, 2017) might occur, and it might work as social support for one of the sources of self-efficacy (Maeda et al., 2013). These suggest that there are different pathways in the effect of the group-based exercise. When examining the effects of this exercise program in the future, it is necessary to comprehensively evaluate not only the psychological symptoms analyzed in this study but also the interactions among psychological and social factors.

This study had some strengths. First, we showed the effectiveness of low-intensity group exercise on subjective insomnia and self-efficacy. Low-dose exercises are tolerable and acceptable for older depressed patients and such exercises should be suggested (Callaghan, Khalil, Morres, & Carter, 2011; Tse, Wong, & Lee, 2015). Although the low-dose exercise group showed better exercise adherence then the high-dose group, investigation of the effectiveness of lighter intensity exercise is sparse (Mura & Carta, 2013). According to a survey conducted by Sakurai et al. (2017) at 1,043 dialysis facilities (88,462 patients) in Japan, about 19% employed physiotherapists and approximately 20% performed exercise programs. However, only 3% of all patients participated in that survey. At present, many patients cannot participate in exercise training. Equipment and personnel availability may be factors affecting the feasibility of exercise programs (Kanamori et al., 2015). Group-based programs include content that allows many patients to benefit from exercise with less specialized staff (Kanamori et al., 2015). Previous studies on community exercise programs for community-dwelling older adults (Benavent-Caballer et al., 2016) and frail older adults (Aida et al., 2007) have shown similar effects, but findings concerning dialysis patients are insufficient. This study’s findings confirm the effectiveness of highly feasible exercise programs in the dialysis room.

This study had some limitations. First, the group assignments were conducted on a cluster basis, and the resulting gender ratio in each cluster differed significantly. Second, although group assignments were quasi-random, because of the nature of the intervention, participants were aware of their group assignment, which may have affected the results. Similarly, although individual effects and missing values were adjusted statistically, it is not possible to determine whether they affected the results. Third, as the data were obtained from only two facilities, the results may not be generalizable to other populations. Fourth, using exercise placebos as a control has been a common practice in studies examining the impact of exercise therapy on depressive symptoms (Dunn et al., 2005; Stubbs et al., 2016). However, because the exercise intensity in the intervention group was lower and those in the control group were higher than predicted. We recorded the RPE of the intervention group only once in each session and did not control the timing of the measurement for practical reasons, which might have affected the results. Additionally, in the group-based resistance exercise using participants’ own weight, which we adopted, the participants might have adjusted their approach to the exercise to make it easier involuntarily. Both the intensity and the effectiveness of resistance training are controversial. A meta-analysis of resistance training’s effect on depression showed that the antidepressant effect of exercise did not depend on a significant improvement in fitness (Gordon et al., 2018). The relationship between intensity of exercise and its physical and psychological effects requires further investigation using, a no-treatment control group (no exercise) is needed. Fifth, the program time for the intervention group was about 30 min, while that for the control group was about 10 min. This difference in program duration may have affected subjective insomnia. Countermeasures to control wake time (e.g. having the control group engage in non-exercise activities like reading for the same amount of time as the intervention group) may be needed in future studies. Finally, this study only measured subjective reports of subjective insomnia. The effects of low-intensity resistance exercise on dialysis patients’ sleep will need to be comprehensively evaluated after conducting studies to assess sleep apnea, restless legs syndrome, sleep latency, and so on.

## 5. Conclusion

A six-month program of low-intensity resistance exercise performed during dialysis improved subjective insomnia and exercise self-efficacy. However, the effect did not maintain. Our findings indicate that long-time follow-up studies are necessary to consider the effect of exercise on mental health. Despite some limitations in the research methodology, this study provided insights into the effects of a low-intensity resistance exercise program that can be performed in daily clinical practice on subjective insomnia and self-efficacy.

## Data Availability

Since the participants of this study did not agree for their data to be shared publicly, supporting data are not available.
